# The endogenous hydrogen gas (H_2_) drives women’s health: a comment on “Gut bacteria convert glucocorticoids into progestins in the presence of hydrogen gas”

**DOI:** 10.3389/fendo.2024.1504814

**Published:** 2025-02-05

**Authors:** Shuangling Yang, Jiongshan Zhang, Luyao Xu, Yajie Guan, Chun Fang, Shuhui Zheng, Hongzhi Yang, Haimei Liu, Yaxing Zhang

**Affiliations:** ^1^ School of Health Sciences, Guangzhou Xinhua University, Guangzhou, Guangdong, China; ^2^ Department of Traditional Chinese Medicine, The Third Affiliated Hospital, Sun Yat-sen University, Guangzhou, Guangdong, China; ^3^ Department of Gynecology, The Second Clinical School of Guangzhou University of Chinese Medicine, The Second Affiliated Hospital of Guangzhou University of Chinese Medicine, Guangdong Provincial Hospital of Chinese Medicine, Guangdong Provincial Academy of Chinese Medical Sciences, Guangzhou University of Chinese Medicine, Guangzhou, Guangdong, China; ^4^ Department of Spleen, Stomach, and Liver and Gallbladder Diseases, The First Affiliated Hospital of Henan University of Chinese Medicine, Zhengzhou, Henan, China; ^5^ College of Animal Science and Technology, Yangtze University, Jingzhou, Hubei, China; ^6^ Research Center for Translational Medicine, The First Affiliated Hospital, Sun Yat-Sen University, Guangzhou, Guangdong, China; ^7^ Department of Physiology, School of Basic Medical Sciences, Guangzhou University of Chinese Medicine, Guangzhou, Guangdong, China; ^8^ Research Centre of Basic Integrative Medicine, School of Basic Medical Sciences, Guangzhou University of Chinese Medicine, Guangzhou, Guangdong, China

**Keywords:** hydrogen gas, 21-dehydroxylation, women health, glucocorticoids, progestins

## 1 Introduction

Hydrogen gas (H_2_), the lightest gas in the universe, can act as an important antioxidant. Supplementation of exogenous H_2_ can improve many diseases. However, the physiological and pathological significance of endogenous H_2_ is not clear. In the recent issue of *Cell*, McCurry et al. reveal that endogenous H_2_ drives 21-dehydroxylation for transforming glucocorticoids to progestins, which may further influence female health, such as postpartum depression (1). We are very interested in this milestone study that reveals the physiological effects of endogenous H_2_. Based on the history, here, we will discuss the process of this important finding, the uncovered questions, the current status, and the future of endogenous H_2_ in female health.

## 2 The gut bacteria chemically modify glucocorticoids into progestins through 21-dehydroxylation

In 1969, Eriksson et al. reported that the feces from germ-free (GF) rats contain corticoids but no pregnanolones, while the feces from conventional rats contain pregnanolones but no corticoids (21-hydroxylated steroids), which suggested that gastrointestinal progestin formation is dependent on gut microbiome (2, 3). Similar to this, it has been reported that fecal slurries from rats and humans can convert glucocorticoids into progestins through reductive removal of a C21 hydroxyl group, a process called 21-dehydroxylation (4, 5). Therefore, this means that the presence of 21-dehydroxylation products in the gut might depend on the gut microbiome. However, it is unclear how gut bacteria chemically modify steroids and how the resulting metabolites affect the host health.

In 2024, Dr. Megan D. McCurry et al. had developed an ultrahigh-performance liquid chromatography (UHPLC)-based method to quantify glucocorticoids and progestins in the biological fluids and tissues (1). Similar to H. Eriksson’s reports (2, 3), total levels of tetrahydroprogesterones (THPs) were significantly reduced in the feces of GF female mice compared with specific-pathogen-free (SPF) female mice (1). Then, they further identify physiologically relevant substrates for 21-dehydroxlation through targeted corticoid profiling on human bile and found that the average concentrations of tetrahydrodeoxycorticosterone (THDOC), e.g., 3α5αTHDOC and 3α5βTHDOC, in the abundant biliary corticoids were higher than other components (1). This indicated that the gut bacteria are exposed to physiologically relevant concentrations of 3α5αTHDOC and 3α5βTHDOC. Because 3α5αTHDOC levels in bile are higher in pregnant women, and its 21-dehydroxylated products have potential biological activities in the context of pregnancy and women’s health (6–8), they next focused on 3α5αTHDOC and hypothesized that 3α5αTHDOC can be 21-dehydroxylated into 3α5αTHP.

To verify their hypothesis, Megan D. McCurry cultured the feces of pregnant GF and SPF mice in the presence of 3α5αTHDOC and measured the levels of progestin, and found that a large amount of 3α5αTHP and 3β5αTHP were produced from pregnant SPF mouse fecal slurries, while fecal slurries from pregnant GF mice did not produce THPs (1). The culture pools with the human feces from healthy females and males 21-dehydroxylated 3α5αTHDOC, and moreover, the feces from pregnant human donors contained substantially higher levels of THPs than the feces from males and non-pregnant females. These indicate that both murine and human gut microbiome have the abilities to 21-dehydroxylate 3α5αTHDOC to produce progestins (1). However, why is there more 21-dehydroxylation product of 3α5αTHDOC in the context of pregnancy? One reason is that 3α5αTHDOC levels in bile are higher in pregnant women (7).

Dr. Megan D. McCurry therefore sought to isolate and characterize the 21-dehydroxylating species from human fecal microbial communities. In a culture pool isolated from a female donor, they found that *Gordonibacter pamelaeae*, a close relative of *Eggerthella lenta*, was the only bacterial species that function as active 21-dehydroxylators (1). Because the abundance of *G. pamelaeae* is low, they used an orthogonal assay and acquired the type strain of this bacterium, *G. pamelaeae* DSM 19378 (1). They found that *G. pamelaeae* DSM 19378 weakly 21-dehydroxylated 3α5αTHDOC to produce 3α5αTHP and 3β5αTHP, and this activity was significantly increased in co-culture with gut commensal *Escherichia coli* Nissle 1917 (*Ec*N); however, *Ec*N alone was unable to perform this transformation (1). They further tested the prevalence of 21-dehydroxylation activity in Eggerthellaceae family. In a 26-strain library of *E. lenta* and *Gordonibacter* species from the human gut, none of these strains performed 21-dehydroxylation in monoculture, and 12 strains produced varying levels of THP in co-culture with *Ec*N (1). Thus, the members of the Eggerthellaceae family 21-dehydroxylate 3α5αTHDOC and indicate that this activity is induced by *Ec*N (1).

## 3 The endogenous H_2_ drives 21-dehydroxylation for transforming 3α5αTHDOC into 3α5αTHP

What are the key mechanisms of induction of 21-dehydroxylation by *E. coli*? It has been reported that 21-dehydroxylation activity is inversely correlated with media redox potential (9). According to this, Dr. Megan D. McCurry first examined the hypothesis that *E. coli* lowers the redox potential and thus promoting 21-dehydroxylation. Their data showed that lower redox potential does not enable 21-dehydroxylation in *E. lenta* 14A (1). Second, they tested whether a 21-dehydroxylation-promoting factor was produced by *EcN*. The physical interactions between microbes can occur during cooperative metabolism; however, they found that contact between the two species is not required to induce 21-dehydroxylation (1). Third, they further tested whether *EcN* released extracellular inducing factors, and they found that *EcN* syringe-filtered supernatants but not vacuum-filtered supernatants induced 21-dehydroxylation in *E. lenta* (1). The basic difference between vacuum filtration and syringe filtration is that the dissolved gases are removed in the former. They therefore hypothesized that *EcN* may produce a gas that leads to 21-dehydroxylation in *E. lenta*. In healthy individuals, the colonic gases are composed of H_2_, carbon dioxide (CO_2_), methane (CH_4_), nitrogen (N_2_), and oxygen (O_2_), as well as several odiferous trace gases, and the former three are produced solely by colonic microbes (10). Thus, which gas or gases could be responsible for 21-dehydroxylation?

It has been reported that *E. coli* can produce H_2_ during its stationary phase of growth (11), which is when Dr. Megan D. McCurry started to observe substantial 21-dehydroxylation. Under anaerobic conditions, H_2_ can be used by microbes as an electron donor to drive sulfate reduction and methanogenesis (12). Moreover, bile acid oxidation by *E. lenta* can be inhibited by H_2_, and *E. lenta* performs reductive metabolic reactions under high H_2_ partial pressure (13). Therefore, they hypothesized that H_2_ might provide the reducing equivalents required for this highly reductive transformation.

**Figure 1:**
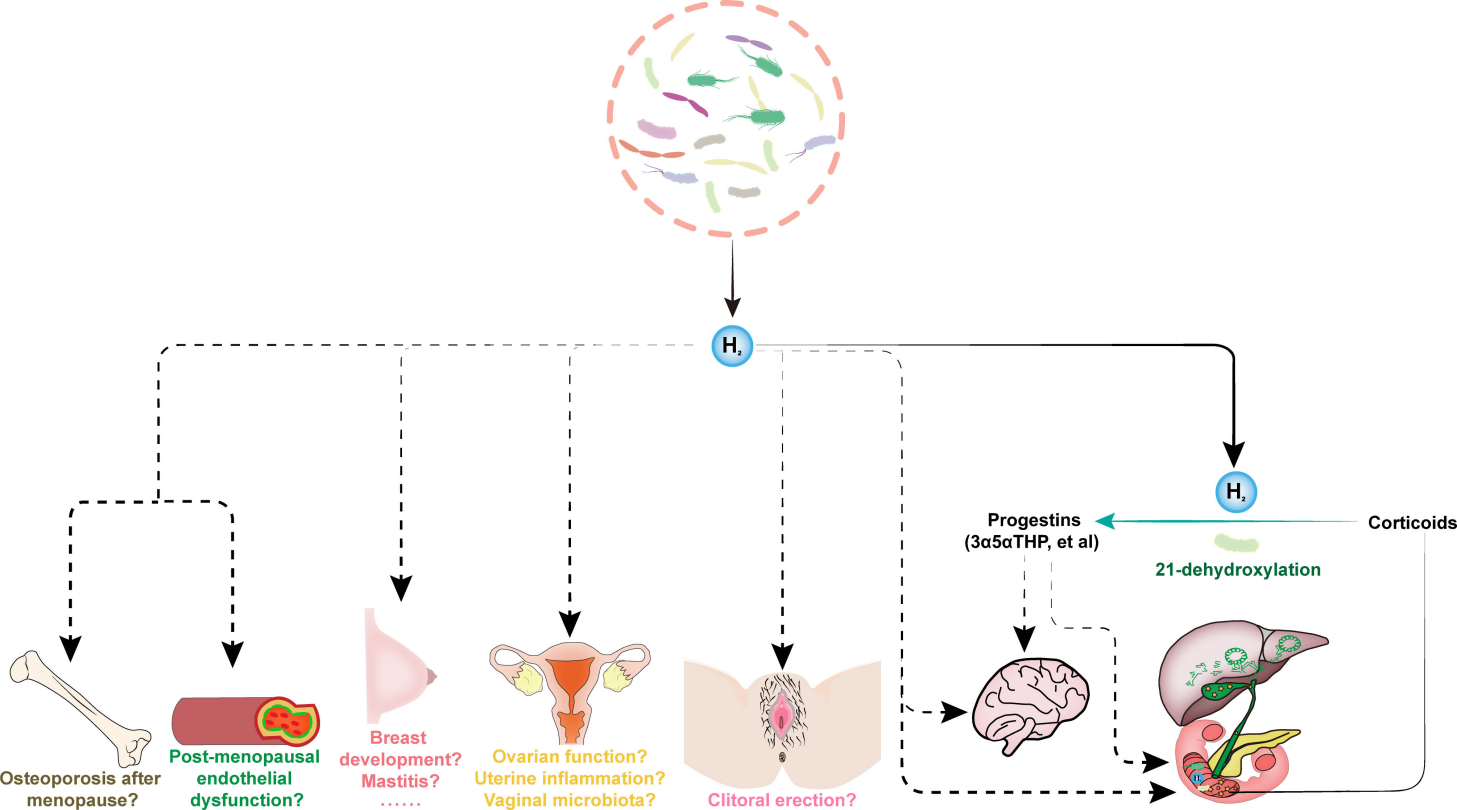
The endogenous H_2_ drives female sexual health. The gut microbiota-derived endogenous H_2_ in female subjects is necessary and sufficient to promote robust 21-dehydroxylation, which is a key step for converting abundant biliary corticoids into progestins, e.g., allopregnanolone (3α5αTHP). The feces from pregnant human donors contained substantially higher levels of THPs than feces from males and non-pregnant females. The low serum levels of 3α5αTHP are related to symptoms of depression in late pregnancy. Allopregnanolone (3α5αTHP), also known as brexanolone, is the first drug approved by the FDA to treat postpartum depression. 3α5αTHP, as a metabolite of gut microbiota, may also have essential impact on gut health. These are indirect effects of endogenous H_2_ dependent on the production of progestins, and endogenous H_2_ may also have direct effects independent on progestins, e.g., affecting intestinal and brain health, among others. The exogenous H_2_ emerged as a novel bioactive molecule involved in modulating sexual organ homeostasis and improving many reproductive diseases in animal models, including erectile dysfunction, polycystic ovary syndrome, chemotherapy-induced ovarian injury, uterine inflammation, mastitis and breast cancer, and postmenopausal osteoporosis. However, the effects of endogenous H_2_ on female health, such as post-menopausal endothelial dysfunction and osteoporosis, normal breast development and mastitis, ovarian function, uterine inflammation, vaginal microbiota, and clitoral erection, are unclear.

Methylene blue (MB) is well known to react with an equimolar amount of H_2_ in the presence of platinum (Pt) or palladium to produce colorless reduced MB (leucomethylene blue, leucoMB), as follows: MB blue + 2H^+^ + 2^e-^ → leucoMB colorless (14). Thus, Dr. Megan D. McCurry used MB with colloidal Pt as H_2_ detection reagent based on the initial work by Tomoki Seo (14) and wildly used by our group (15) and others (16, 17). They found that *E. lenta* 14A grown under a H_2_ condition, rather than a N_2_ condition, performed 21-dehydroxylation. The H_2_ levels and 21-dehydroxylation activities of *E. coli* with hydrogenase mutations co-cultured with *E. lenta* 14A were more than four times lower than those of *E. coli* BW25113 co-cultured with *E. lenta* 14A (1). In contrast to this, the H_2_ levels and 21-dehydroxylation activities were not significantly influenced by disrupting a reductive pathway unrelated to H_2_ production through loss of *cysJ*, the NADH:flavin oxidoreductase for the sulfite reductase in *E. coli*, when co-cultured with *E. lenta* 14A (1). Thus, H_2_ produced by *E. coli* is a major driving force that promotes robust 21-dehydroxylation in *E. lenta* (**Figure 1**). Beyond *Ec*N 1917, the Gram-positive strains *Clostridium scindens* VPI 12780 and *C. perfringens* ATCC 13124, and the Gram-negative strains *Citrobacter rodentium* ATCC 8090 and *C. freundii* ICC 168, which express hydrogenase or hydrogenase homologs, can also induce 21-dehydroxylation in *E. lenta* 14A (1).

It should be noted that 21-dehydroxylation was not reduced to undetectable levels in co-cultures with H_2_-deficient mutants, indicating that other factors produced by EcN may also contribute to this reaction (1). Their data also showed that there are low levels of H_2_ produced in the co-culture of *E. lenta* 14A and *E. coli* with hydrogenase mutations (1). Thus, where do they come from? Indeed, *E. lenta* also contains annotated but unconfirmed hydrogenases, and they found that the *E. lenta* 14A monoculture can produce levels of H_2_ comparable to those of the *E. coli* Nissle monoculture (1). In another independent experiment, Megan D. McCurry found that the *E. lenta* 14A monoculture and *G. pamelaeae* DSM 19743 monoculture produce the same levels of H_2_ (1). 3α5αTHP and 3β5αTHP were only detected in the *G. pamelaeae* DSM 19743 monoculture, but not in the *E. lenta* 14A monoculture (1). This indicated that *G. pamelaeae* DSM 19743 may contain or produce other factors, rather than H_2_, to induce 21-dehydroxylation. The synergistic H_2_ production and 21-dehydroxylation of co-culture by *E. lenta* 14A and *Ec*N 1917, and by *G. pamelaeae* DSM 19743 and *Ec*N 1917, were higher than the monoculture of *E. lenta* 14A (ND) or *G. pamelaeae* DSM 19743 (1). Therefore, they concluded that higher H_2_ produced in co-culture promoted 21-dehydroxylation activity.

Thus, which candidate genes could be involved in 21-dehydroxylation in *E. lenta*? They performed comparative genomics analysis on the genome sequences of producer and non-producer strains from the Actinobacteria strain library, and identified gene cluster Elen_2451–Elen_2454 as the candidate genes for 21-dehydroxylation (1). By co-culturing one of the top producer strains, *E. lenta* 14A, with EcN with or without tungstate, which is known to inhibit the oxidoreductase activity of Elen_2453, and by homologously and heterologously expressing the Elen_2451–2454 cluster, they confirmed that Elen_2451–2454 is responsible for 21-dehydroxylation in *E. lenta* (1). The feces from pregnant subjects are enriched in Elen_2451–2454 cluster-containing bacteria, and the concentrations of THPs positively correlated with the abundance of Elen_2451–2454 homologs (1). The feces from co-colonized female GF mice with producer strain *E. lenta* 14A and EcN contained significantly more THPs than female GF controls (1). Moreover, the co-colonized GF female mice with EcN and the cluster-containing strain *E. lenta* 14A, rather than the cluster-lacking strain *E. lenta* A2, produced higher levels of total THPs and 3α5βTHP in the feces (1). Elen_2451 is a formate dehydrogenase family accessory protein FdhD, Elen_2452 is 4Fe-4S ferredoxin iron-sulfur binding domain protein, Elen_2453 is molybdopterin oxidoreductase, and Elen_2454 is an SPFH domain band 7 family protein (1). However, we are still not clear about enzymatic mechanisms by which the above cluster regulate 21-dehydroxylation.

## 4 Discussion: there is still a long way to go for endogenous H_2_ in female health

The ovarian hormone (progesterone and estrogen) levels increase over 100-fold during pregnancy, and consequently, neurosteroid allopregnanolone (also as brexanolone, 3α5αTHP) is also elevated in the brain (18, 19). The GABA_A_R expression in the brain is reduced by neurosteroids during pregnancy to prevent sedation (18–20). Upon parturition, hormones rapidly return to pre-pregnancy levels, but the expression of GABA_A_R may take time to recover (19). Therefore, a long-lasting mismatch between neurosteroid levels and GABA_A_R numbers may underlie postpartum depression (19). The serum levels of allopregnanolone were detectable postpartum and were significantly decreased in women with maternity “blues” (8). Brexanolone is a first Food and Drug Administration (FDA)-indicated drug for postpartum depression (21). The gut bacteria both consume and produce H_2_, and the remaining net H_2_ in a fecal culture from donor F2 was sufficient to complete 21-dehydroxylation in co-culture and produced THPs (1). The THPs, such as 3α5αTHP, 3α5βTHP, 3β5αTHP, and 3β5βTHP, are two orders of magnitude higher in feces from pregnant people in the third trimester compared with the feces of male and nonpregnant female subjects (1). However, we are unclear about the changes in levels of gastrointestinal-derived THPs in pregnant women with postpartum depression before and after delivery. We do not know whether progestins produced by *E. lenta* remain in the gastrointestinal tract or are absorbed into enterohepatic or systemic circulation (1). Thus, it is not clear whether gut-derived THPs can affect postpartum depression (**Figure 1**). Supplementation of exogenous H_2_ protected against depression in mice (22), and if THPs can be absorbed into enterohepatic or systemic circulation and have effects on depression, it is difficult to distinguish the observed effects of exogenous H_2_ on depression that are caused directly by H_2_ or indirectly by produced THPs or by the synergistic effects of H_2_ and THPs (**Figure 1**).

Sex entails cutting-edge science but is bathed in mystery, and it is a fundamental pleasure and quality-of-life issue (23). H_2_ has emerged as a novel bioactive molecule involved in modulating sexual organ homeostasis (23). Supplementation of exogenous H_2_ can improve erectile dysfunction in a model of diabetic rats (24) and alleviate polycystic ovary syndrome (25), chemotherapy-induced ovarian injury (26), uterine inflammation (27), mastitis (28) and breast cancer (29, 30), and postmenopausal osteoporosis (31). It also has the effect of vasodilation, and attenuates chronic intermittent hypoxia-induced hypertension in rats (32, 33). However, the effects of endogenous H_2_ on female health, such as post-menopausal endothelial dysfunction, osteoporosis after menopause, normal breast development and mastitis, ovarian function, uterine inflammation, vaginal microbiota, and clitoral erection are unclear (**Figure 1**). To answer the effects of endogenous H_2_ on female health, we should confirm biological distributions of endogenous H_2_ (and its related metabolites, such as THPs) produced by microbiota, and analyze the changes of endogenous H_2_ (and its related metabolites, such as THPs) and microbiota in blood and feces and organs/tissues before and after disease (or compare between normal individuals and patients). The animal models should be used to answer the causal relationship between endogenous H_2_ and female health. Moreover, the strategies to reduce endogenous H_2_ and increase endogenous H_2_ should be employed. Reducing endogenous H_2_ is typically achieved by the systematic use of antibiotics; however, the effects of antibiotics are broad and not specific, and both beneficial and harmful bacteria will be affected; therefore, this indiscriminate treatment cannot accurately answer the effects of endogenous H_2_. After killing gut microbial community via antibiotics, it seems that supplementing with indicated strains can provide a relatively accurate answer to the physiological functions of indicated bacteria (34). We should note that it is not possible to have only one type of bacteria in the body, and the indicated bacteria can interact with one or more other bacteria or the host cells in multiple dimensions, thereby producing a range of physiological effects.

Although there are many challenges in H_2_ medicine, especially endogenous H_2_, Megan D. McCurry’s study provides a new paradigm for future research on H_2_ medicine. Based on the expression profile of hydrogenase, we should investigate the physical and chemical essences of production and utilization of H_2_ by human microorganisms and the physical and chemical essences of utilization of H_2_ by the host cells and, thus, confirm the significance of endogenous H_2_ for human homeostasis and pathogenesis of diseases.
